# Hypoxia-induced mitogenic factor (HIMF/FIZZ1/RELMα) in chronic hypoxia- and antigen-mediated pulmonary vascular remodeling

**DOI:** 10.1186/1465-9921-14-1

**Published:** 2013-01-04

**Authors:** Daniel J Angelini, Qingning Su, Kazuyo Yamaji-Kegan, Chunling Fan, John T Skinner, Andre Poloczek, Hazim El-Haddad, Chris Cheadle, Roger A Johns

**Affiliations:** 1 Department of Anesthesiology and Critical Care Medicine, Johns Hopkins University School of Medicine, 720 Rutland Avenue, Ross 361, Baltimore, MD 21205, USA; 2 Division of Cardiology, Department of Medicine, Johns Hopkins University School of Medicine, Baltimore, MD, 21205, USA; 3 Division of Allergy and Clinical Immunology, Department of Medicine, Johns Hopkins University School of Medicine, Baltimore, MD, 21205, USA; 4 Division of Pulmonary and Critical Care Medicine, Department of Medicine, Johns Hopkins University School of Medicine, Baltimore, MD 21205, USA

**Keywords:** Pulmonary hypertension, Hypoxia-induced mitogenic factor (HIMF), Chronic hypoxia, Th2-mediated inflammation, Vascular remodeling

## Abstract

**Background:**

Both chronic hypoxia and allergic inflammation induce vascular remodeling in the lung, but only chronic hypoxia appears to cause PH. We investigate the nature of the vascular remodeling and the expression and role of hypoxia-induced mitogenic factor (HIMF/FIZZ1/RELMα) in explaining this differential response.

**Methods:**

We induced pulmonary vascular remodeling through either chronic hypoxia or antigen sensitization and challenge. Mice were evaluated for markers of PH and pulmonary vascular remodeling throughout the lung vascular bed as well as HIMF expression and genomic analysis of whole lung.

**Results:**

Chronic hypoxia increased both mean pulmonary artery pressure (mPAP) and right ventricular (RV) hypertrophy; these changes were associated with increased muscularization and thickening of small pulmonary vessels throughout the lung vascular bed. Allergic inflammation, by contrast, had minimal effect on mPAP and produced no RV hypertrophy. Only peribronchial vessels were significantly thickened, and vessels within the lung periphery did not become muscularized. Genomic analysis revealed that HIMF was the most consistently upregulated gene in the lungs following both chronic hypoxia and antigen challenge. HIMF was upregulated in the airway epithelial and inflammatory cells in both models, but only chronic hypoxia induced HIMF upregulation in vascular tissue.

**Conclusions:**

The results show that pulmonary vascular remodeling in mice induced by chronic hypoxia or antigen challenge is associated with marked increases in HIMF expression. The lack of HIMF expression in the vasculature of the lung and no vascular remodeling in the peripheral resistance vessels of the lung is likely to account for the failure to develop PH in the allergic inflammation model.

## Background

Pulmonary hypertension (PH) is clinically defined by a mean pulmonary artery pressure (mPAP) ≥ 25 mmHg at rest [1,2]. This condition can manifest itself as a primary disease (idiopathic with or without a genetic linkage) or as a complication of another disease such as collagen vascular disease (e.g., scleroderma), human immunodeficiency virus (HIV) infection, *Shistosoma mansoni* (*S. mansoni*) infection, or chronic obstructive pulmonary disease [1-4]. The exact initiating event in the pathogenesis of PH is largely unknown, but several conditions, including chronic hypoxia and chronic pulmonary inflammation, have been associated with the development of PH in humans [5-7]. These conditions induce endothelial cell dysfunction as well as endothelial and vascular smooth muscle cell proliferation and hypertrophy within the pulmonary circulation [8]; changes in the cells of the vasculature thicken pulmonary arteries and arterioles, narrowing their lumens, resulting in increases in pulmonary vascular pressure and resistance [5,8,9].

A growing body of evidence suggests that inflammation plays a role in the vascular remodeling associated with chronic hypoxia and other experimental manifestations of PH [10-14]; these findings have accelerated investigation into the potential mechanisms of inflammation in this disease process [7]. Burke *et al.*[11] reported the upregulation of several pro-inflammatory genes in the lungs, including stromal cell-derived factor-1 (SDF-1/CXCL12), monocyte chemoattractant protein-1 (MCP-1), and interleukin (IL)-6, as well as the accumulation of monocytes within the pulmonary vasculature during chronic hypoxia. Also, many studies have shown that T-cell helper 2 (Th2) cytokines, particularly IL-4 and IL-13, are involved in the pathogenesis of pulmonary vascular remodeling [13-18]. We have recently reported that IL-4 acts as a proangiogenic molecule in the lungs of mice during chronic hypoxia [13]. To examine the role of Th2 cytokines in pulmonary vascular remodeling, Daley *et al.*[15] modified the standard antigen-challenge model normally used to examine inflammatory asthma. In this modified model, extended antigen challenges with either ovalbumin (Ova) or *Aspergillus fumigatus* antigen (*Asp* ag) lacking any viable fungus produced severe pulmonary vascular remodeling involving the proliferation of vascular smooth muscle cells. This remodeling was reduced in both IL-4 knockout mice and mice that had IL-13 signaling neutralized [15]. Surprisingly, this model produced no increases in right ventricular systolic pressure. In an *S. mansoni* infection model of PH, Graham *et al.*[19] demonstrated that mice that lacked the IL-13 decoy receptor, IL-13Rα2, displayed more severe pulmonary vascular remodeling than did wild-type controls. They also reported an increase in right ventricle (RV) maximum pressure in the infected IL13Rα2 knockout mouse compared to uninfected wild-type controls. In a separate study, Swain *et al.*[17] reported pulmonary vascular remodeling and development of PH as a result of *Pneumocystis* pneumonia in both wild-type and CD4^+^ T-cell-depleted mice; notably, these pathological changes still occurred in IL-4 knockout mice, and IL-13 was not detected in the lungs of the mice during the persistent phase of the model [17]. These studies suggest a role for inflammation in pulmonary vascular remodeling, but currently, the exact involvement in this process is unclear.

Hypoxia-induced mitogenic factor (HIMF), also known as “found in inflammatory zone 1” (FIZZ1) or “resistin-like molecule alpha” (RELMα), is highly upregulated in the lung in response to both chronic hypoxia [10,20,21] and Th2-mediated inflammation [16,17,22-27]. We have demonstrated that HIMF has proliferative, angiogenic, vasoconstrictive, and chemokine-like properties that are associated with the development of PH [10,20,21,28,29]. We have also demonstrated that overexpression of HIMF within the lungs induces a pattern of vascular remodeling and hemodynamic changes similar to that in chronic hypoxia-induced PH and that the *in vivo* blockade of HIMF expression within the lung reduces the pathologic vascular and hemodynamic changes associated with this model [10,20]. These data indicate that HIMF plays a direct role in the induction of pulmonary vascular remodeling and the development of PH associated with chronic hypoxia. HIMF is also upregulated in response to pulmonary inflammation [16,17,22-25,27,30]. It has been reported that HIMF expression is increased in the lungs of several models of Th2-dependent inflammation, including allergic asthma [15,22,23,27], human herpes virus 8 infection [25], *Pneumocystis* pneumonia [17], *S. mansoni* infection [16,19], and bleomycin-induced pulmonary fibrosis [24,30]; all of these models are associated with pulmonary vascular remodeling. Our laboratory has demonstrated that a tail vein injection of recombinant murine HIMF into mice induces a pro-inflammatory state within the lungs associated with vascular remodeling [14] and that HIMF can induce *de novo* production of both SDF-1 and MCP-1 in cultured endothelial cells and *ex vivo* lung organ culture [14,29]. We have also shown that the human isoform of HIMF, RELMβ, is upregulated in the lungs of patients diagnosed with scleroderma-associated PH [31]. In lung samples from these patients, RELMβ was expressed in inflammatory cells (macrophages, T-cells) as well as in myofibroblasts, endothelium, and vascular smooth muscle [31]. Renigunta *et al.*[32] also demonstrated that hypoxia could induce human RELMβ expression in both a lung cancer cell line (A549) and primary pulmonary artery smooth muscle cells *in vitro*.

At the current time, it remains unclear whether Th2 models of vascular remodeling induction, including Ova, *Asp* ag, and *S. mansoni* infection, actually cause the development of PH as chronic hypoxia does (e.g. increased mPAP, RV hypertrophy, vascular remodeling). In the current study, we directly compare chronic hypoxia- and Th2 inflammation-induced pulmonary vascular remodeling to address this issue and identify possible explanations of the observed differences.

## Methods

### Experimental animals

Adult male C57BL/6 mice (6–8 weeks old; Charles River Laboratories, Wilmington, MA) were used for all of the studies. The animal housing and experimental protocols were approved by the Animal Care and Use Committee of the Johns Hopkins University. The mice had free access to food and water and were housed in a room with a 12:12 h light–dark cycle at 20–24°C.

### Ova model of Th2-induced pulmonary vascular remodeling

The Ova used for this study was prepared as follows: Ova (Grade V; Sigma-Aldrich, St. Louis, MO) was diluted to 1 mg/ml in 0.15 M sterile saline that was complexed with Alum (Imject Alum; Thermo Fisher Scientific, Waltham, MA). Mice were injected i.p. with 50 μg Ova and 2 mg Alum or equivalent volume of sterile saline solution on day 0 of the experiment. This procedure was then repeated on day 14. On days 28–30, the mice were intranasally challenged with 50 μg Ova diluted in 50 μl of saline or with 50 μl saline alone. The intranasal challenges were also repeated on days 35–37, 41–43, and 45. Mice were sacrificed on day 46 of the experiment by isoflurane overdose, and tissue was processed as we have described [10,20]. Mice used for hemodynamic measurement were anesthetized with an i.p. injection of ketamine (100 mg/kg) and xylazine (10 mg/kg), and mPAP was determined as described [20,21]. All mice were euthanized by exsanguination. Bronchoalveolar lavage fluid (BALF) was collected by injecting the airways with 0.8 ml of sterile saline solution under constant pressure via a 22-gauge catheter inserted into the trachea followed by removal under constant pressure. The BALF was centrifuged to remove cells and cell particles; the supernatant was stored at −80°C for use in Western blotting. The heart and lungs from each mouse were removed en bloc. The right lung was tied off, and the left lung was inflated with 1% low-melt agarose in PBS with constant pressure (25 cm H_2_O) and placed on ice as we have described previously [10,20]. The right lung was removed and frozen in liquid nitrogen and stored at −80°C for use in Western blotting. The heart was then removed and the agarose-inflated left lung was placed in 10% neutral buffered formalin (Thermo Fisher Scientific) at 4°C for 48–72 h. Following fixation, the left lung was processed for paraffin embedding. The heart was then dissected into the RV and left ventricle plus septum (LV+S). Both portions of the heart were then weighed and the RV and LV+S ratio determined [RV/(LV+S)].

### Asp ag model of Th2-induced pulmonary vascular remodeling

*Asp* ag that did not contain any viable fungus (GREER Laboratories, Inc., Lenoir, NC) was diluted to 1 mg/ml in sterile saline. Mice were injected i.p. with 100 μl of this solution on days 0 and 14 of this experiment. On days 28–30, the mice were intranasally challenged with 100 μg *Asp* ag diluted in 50 μl of saline or with 50 μl saline alone. The intranasal challenges were also repeated on days 35–37, 41–43, and 45. On day 46, the mice were sacrificed and processed as stated above.

### Chronic hypoxia model of pulmonary vascular remodeling

Mice were exposed to normal room air (20.8% O_2_) or 10.0% O_2_ for 4 or 28 days as we have described [10,20,21,33-36]. The mice were housed in Plexiglas hypoxia chambers that had continuous air flow. The fractional concentration of O_2_ was monitored and controlled with a Pro:Ox model 350 unit (Biospherix, Redfield, NY) by infusion of N_2_ (Roberts Oxygen; Rockville, MD) balanced against an inward leak of air through holes in the chamber. CO_2_ and ammonia were scavenged throughout the experiment. At each time point, the mice were sacrificed and processed as stated above.

### Assessment of pulmonary vascular remodeling

Pulmonary vascular remodeling was assessed in a blinded fashion as we have previously described [10,20,33-36]. For our initial evaluation, lung sections were stained with hematoxylin and eosin. To evaluate the muscularization of pulmonary vessels, we dual stained additional lung sections for von Willebrand Factor (endothelium) and α-smooth muscle actin (vascular smooth muscle) as we have previously described [10,20,35,36]. We then examined approximately 100 randomly selected peripheral vessels (<80 μm in diameter) from each mouse (n ≥ 3 for each experimental group) under an Olympus-BHS microscope attached to a QImaging Retiga 4000RV digital camera. These vessels were classified as non-muscular (NM), partially muscular (PM), or fully muscular (FM) and analyzed as we have published [10,20]. Vascular remodeling was also evaluated by the percent of medial thickness (%MT) of pulmonary vessels as described [20,33]. Briefly, %MT was determined on the murine lung sections receiving Movat pentachrome stain and calculated as follows: %MT = [(external diameter – internal diameter)/external diameter] × 100. For these studies, we evaluated vessels that had an external diameter between 25 and 150 μm. Approximately 30 vessels in 30 consecutive fields were evaluated per lung. Pulmonary vessels were classified as either peribronchial or intra-alveolar. MetaMorph software (Molecular Devices, Downingtown, PA) was used to make the measurements.

### Western blot analysis

Frozen mouse lung tissue was homogenized on ice with a Brinkman Homogenizer (Polytron, Westbury, NY) in homogenization/lysis buffer and the resultant protein assayed as we have described [20]. The lung and BALF samples were resolved by a 4–20% SDS-PAGE (Bio-Rad, Hercules, CA) gel and transferred onto nitrocellulose (Bio-Rad) membranes. The membranes were blocked with 5% milk in Tris-buffered saline with 0.1% Tween 20 (TBS-T) for 1 h and then incubated with polyclonal goat anti-mouse RELMα (1:500; R&D Systems, Minneapolis, MN) antibodies in 5% milk/TBS-T overnight at 4°C. The blots were then incubated in rabbit anti-goat IgG antibodies (1:5000; Bio-Rad) conjugated to horseradish peroxidase in 5% milk/TBS-T for 1h at room temperature. Enhanced chemiluminescence (Amersham, Piscataway, NJ) was used to develop the blots followed by exposure to X-ray film (Denville Scientific, Metuchen, NJ). To ensure equal loading and transfer, blots were stripped with Blot Restore Membrane Rejuvenation Kit (Millipore) and reprobed with murine anti-β actin (Santa Cruz Biotechnology, Santa Cruz, CA).

### Immunohistochemistry

Paraffin blocks of murine lungs exposed to saline challenge, Ova challenge, normoxia (20.8% O_2_), or hypoxia (10.0% O_2_) were cut into 6-μm sections and placed on clean positively charged glass slides. We deparaffinized, rehydrated, and performed antigen retrieval on the slides as described [10,20]. Endogenous peroxidases, avidin, and biotin were blocked as described [10,20]. Nonspecific protein binding was blocked by incubating the sections in normal rabbit serum for 1 h at room temperature. Then, the sections were treated with polyclonal goat anti-mouse RELMα (1:200; R&D Systems, Minneapolis, MN) antibodies or antibody diluent alone overnight at 4°C. After being washed with PBS, the slides were treated with rabbit anti-goat biotinylated secondary antibodies and then an ABC horseradish peroxidase reagent (Vectastain Elite ABC Kit; Vector Laboratories) for 30 min each at room temperature. The HIMF signal was developed with the Peroxidase Substrate Kit DAB (Vector Laboratories), counterstained with hematoxylin, dehydrated, and mounted as we have described [10,20]. The signal was visualized with an Olympus-BHS microscope attached to a QImaging Retiga 4000RV digital camera. Images were captured with ImagePro Plus (version 5.1) software.

### Immunofluorescence microscopy

Paraffin-embedded murine lung tissue was sectioned, deparaffinized, and rehydrated; antigen retrieval was performed as described under *Immunochemistry*. The slides were then washed with PBS, and nonspecific protein binding was blocked by incubating the sections in normal horse serum for 1 h at room temperature. The sections were then treated with polyclonal rabbit anti-mouse RELMα (1:200; Abcam, Cambridge, MA) and mouse anti-α-smooth muscle actin (1:500), rat anti-F4/80 (1:100; Serotec, Raleigh, NC), mouse anti-CD3 (1:200; Santa Cruz Biotechnology), rat anti-major basic protein (MBP; 1:500; Santa Cruz Biotechnology), rat anti-neutrophil (NIMP-R14; 1:100; Abcam), rat anti-CD19 (1:200; Abcam), or diluents alone overnight at 4°C. Next, the sections were incubated with DyLight 488-labeled goat anti-rabbit IgG (H+L) (1:200; Jackson ImmunoResearch Labs, Inc., West Grove, PA) and either DyLight 594-labeled donkey anti-mouse IgG (H+L) (1:200; Jackson ImmunoResearch Labs, Inc.) or DyLight 594-labeled donkey anti-rat IgG (H+L) (1:200; Jackson ImmunoResearch Labs, Inc.) for 45 min at room temperature in the dark. Finally, the sections were washed in PBS, mounted with Vectashield Hardset Mounting Media with 4’,6’-diamidino-2-phenylindole dilactate (DAPI; Vector Laboratories), covered with a glass coverslip, and visualized with an Olympus-BHS microscope attached to a QImaging Retiga 4000RV digital camera. Images were captured by using ImagePro Plus (version 5.1) software and colorized with Adobe Photoshop CS5 software (Adobe Systems, Inc., San Jose, CA).

### Genomic analysis

RNA was extracted from lung tissue with the Trizol Reagent method (Invitrogen, Carlsbad, CA). Additional purification was performed on RNAeasy columns (Qiagen, Valencia, CA). The quality of total RNA samples was assessed by using an Agilent 2100 Bioanalyzer (Agilent Technologies, Palo Alto, CA). The RNA samples were labeled according to the chip manufacturer’s recommended protocols. Briefly, 0.5 μg of total RNA from each sample was labeled by using the Illumina TotalPrep RNA Amplification Kit (Ambion, Austin, TX) in a two-step process of cDNA synthesis followed by *in vitro* RNA transcription. Single stranded RNA (cRNA) was generated and labeled by incorporating biotin-16-UTP (Roche Diagnostics GmbH, Mannheim, Germany). Seven hundred fifty nanograms of biotin-labeled cRNA was hybridized for 16 h to Illumina Sentrix Mouse Ref8_v1.1 BeadChips (Illumina, San Diego, CA). The hybridized biotinylated cRNA was detected with streptavidin-Cy3 and quantified by using Illumina’s BeadStation 500GX Genetic Analysis Systems scanner. Preliminary analysis of the scanned data was performed with Illumina BeadStudio software, which returns single intensity data values/gene following the computation of a trimmed mean average for each probe type represented by a variable number of bead probes/gene on the array. Z-transformation for normalization was performed on each Illumina sample/array on a stand-alone basis [37], and significant changes in gene expression between class pairs were calculated by Z test [38]. Significant gene lists were calculated by selecting genes that satisfied a significance threshold criteria of Z test *P* values ≤ 0.001, a false discovery rate ≤ 0.1 [39], and a fold change ± 1.5 or greater. Changes in gene expression across multiple groups were tested for by one-way ANOVA. Gene expression signatures robustly identified on the basis of statistical significance were tested against multiple sample datasets by using a systematic gene set analytical approach such as Parametric Analysis of Gene Expression (PAGE) [40] or Gene Set Enrichment Analysis [41]. Differential regulation of related gene expression signatures were quantified and compared by using Gene Set Matrix Analysis [42], a modification of the PAGE method in which multiple gene lists were automatically tested against one or more datasets for parametric enrichment (using the log ratios of calculated changes in gene expression for the entire dataset).

### Statistical analysis

Experimental values are expressed as means ± standard error of the mean (SEM). A paired Student’s *t*-test was used to compare the mean responses between two groups. ANOVA was used to compare the mean responses between experimental and control groups for experiments containing multiple groups. The Tukey multiple comparison test was used to determine between which groups significant differences existed. A *P* value of <0.05 was considered statistically significant.

## Results

### Induction of pulmonary vascular remodeling by chronic hypoxia and Th2 inflammation

**Figure 1:**
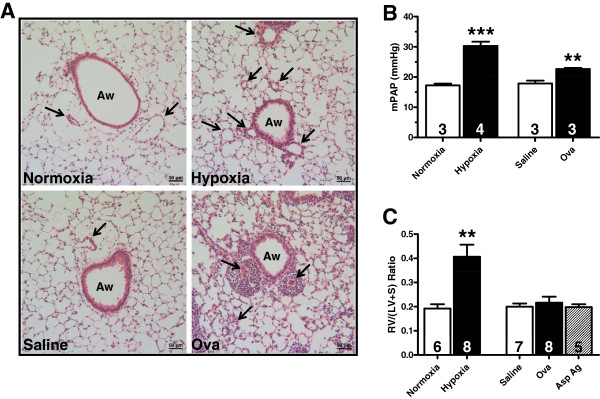
**Chronic hypoxia- and antigen-induced pulmonary vascular remodeling.** (**A**) Paraffin-embedded lung sections from normoxic (28 days, 20.8% O_2_), hypoxic (28 days, 10.0% O_2_), saline-challenged, and ovalbumin (Ova)-challenged mice were rehydrated and stained with hematoxylin and eosin. Arrows: pulmonary vessels. Aw: airway. Scale bar: 50 μm. (**B**) Mean pulmonary artery pressure (mPAP) in mice exposed to normoxia, hypoxia, saline challenge, and Ova challenge. (**C**) Ratio of right ventricular (RV) weight to left ventricular plus septum (LV+S) weight [RV/(LV+S)] in mice exposed to normoxia, hypoxia, saline challenge, Ova challenge, and *Aspergillus fumingatus* antigen (*Asp* ag) challenge. Data are shown as means ± SEM. The number of mice studied is indicated within each bar. **P<0.01, ***P<0.001 versus control.

Mice exposed to chronic hypoxia exhibited increased muscularization and thickening of the pulmonary vasculature as well as mild inflammation, when compared to normoxic controls (Figure 1A). The Ova-challenged mice displayed vascular remodeling mainly around the airways. In addition, massive numbers of inflammatory cells surrounded and invaded the adventitia of the remodeling blood vessels. Mice challenged with *Asp* ag displayed a similar pattern of vascular remodeling and inflammation to that of Ova-challenged mice (data not shown). Saline-challenged mice displayed normal pulmonary histology (Figure 1A).

### Effect of chronic hypoxia and Th2 inflammation on indicators of PH

Mice exposed to chronic hypoxia for 28 days had an elevated mPAP (~1.75-fold) compared to normoxic controls (hypoxia: 30.29 ± 1.44 mmHg vs. normoxia: 17.23 ± 0.57 mmHg; *P*<0.001; Figure 1B). Further analysis revealed that animals exposed to hypoxia had a ~2.1-fold increase in RV/(LV+S) ratio (0.407 ± 0.050) compared to normoxic controls (0.192 ± 0.018; Figure 1C). In a separate experiment, mice that were challenged with intranasal Ova exhibited a slight (~1.3-fold) increase in mPAP compared to mice challenged with saline (Ova challenge: 22.65 ± 0.37 mmHg vs. saline: 17.88 ± 0.94 mmHg; *P*<0.01; Figure 1B). Even though mPAP increased slightly in the Ova-challenged mice, there were no changes in RV/(LV+S) ratio when compared to saline-challenged controls (Ova challenge: 0.216 ± 0.025; saline: 0.200 ± 0.013; Figure 1C). *Asp* ag challenge also failed to produce a change in RV/(LV+S) ratio (0.198 ± 0.012; Figure 1C).

### Evaluation of chronic hypoxia- and Th2 inflammation-induced pulmonary vascular remodeling

**Figure 2:**
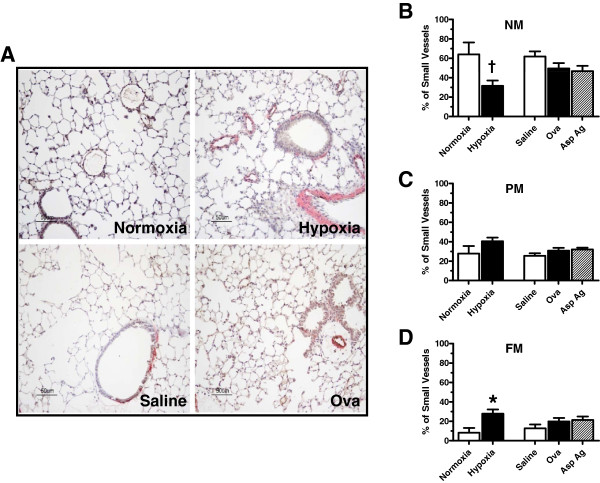
**Evaluation of chronic hypoxia- and antigen-induced muscularization of small pulmonary vessels.** (**A**) Paraffin-embedded lung sections from normoxic (28 days, 20.8% O_2_), hypoxic (28 days, 10.0% O_2_), saline-challenged, Ova-challenged, and *Asp* ag-challenged mice were rehydrated and double-stained with antibodies to von Willebrand factor (black) and α-smooth muscle actin (red). Sections were counterstained with hematoxylin (blue). Scale bar: 50 μm. (**B-D**) Percent muscularization of small pulmonary vessels in mouse lung. NM, non-muscularized (**B**); PM, partially muscularized (**C**); FM, fully muscularized. n ≥ 3 mice for each group. ^†^Significantly decreased versus control at *P*<0.05. *Significantly increased versus control at *P*<0.05.

In mice exposed to chronic hypoxia, muscularization of the small pulmonary vessels was increased over the course of the experiment (Figure 2). After exposure to 10.0% O_2_ for 28 days, the percentage of FM vessels was increased (27.76 ± 4.48% vs. 8.25 ± 3.37%; *P*<0.05; Figure 2D), while the percentage of NM vessels decreased (31.58 ± 5.49% vs. 64.00 ± 12.28%; *P*<0.05) compared to control animals exposed to normoxia (Figure 2B). Examination of the lung sections from Ova-challenged mice revealed no significant increases in new muscularization (Figure 2B–D). Similar results were observed following *Asp* ag challenge (NM: 46.72 ± 5.49%: PM: 32.71 ± 3.14%; FM: 21.26 ± 3.66%). Histological representation of tissue from mice exposed to chronic hypoxia and Ova challenge is shown in Figure 2A.

**Figure 3:**
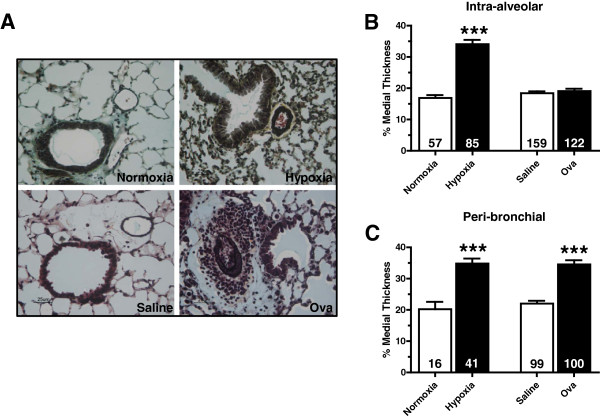
**Evaluation of chronic hypoxia- and Ova-induced increases in percent of medial thickness (%MT) in pulmonary vessels.** (**A**) Paraffin-embedded lung sections from normoxic (28 days, 20.8% O_2_), hypoxic (28 days, 10.0% O_2_), saline-challenged, and Ova-challenged mice were rehydrated and stained with Movat’s pentachrome stain. Scale bar: 25 μm. Intra-alveolar (**B**) and peribronchial (**C**) %MT in mice exposed to normoxia, hypoxia, saline challenge, and Ova challenge. Data are shown as means ± SEM. The number of vessels counted is indicated within each bar. n ≥ 4 mice for each group. ***P<0.001 versus control.

The %MT of pulmonary vessels was increased in mice exposed to chronic hypoxia compared to that of normoxic controls (Figure 3). Hypoxia increased the %MT of intra-alveolar vessels from 16.67 ± 0.90% to 34.29 ± 1.60% (Figure 3B) and of peribronchial vessels from 20.23 ± 2.36% to 34.81 ± 1.60% (Figure 3C). Hypoxia increased the thickness of both types of vessels in a similar fashion. Although Ova-challenged mice did not display significant new muscularization of small pulmonary arteries, these challenges had a profound effect on hypertrophy/hyperplasia of the already muscularized peribronchial vessels within the lung (Figure 3C). When vessels associated with large airways were compared, %MT increased from 22.01 ± 0.92% in saline controls to 34.53 ± 1.35% following Ova challenge, increases very similar to those observed in pulmonary vessels from hypoxia-exposed mice. Ova challenge had virtually no effect on the %MT of intra-alveolar vessels (Ova: 19.09 ± 0.77% vs. saline: 18.43 ± 0.58%; Figure 3B).

### Genomic analysis of lung tissue

**Figure 4:**
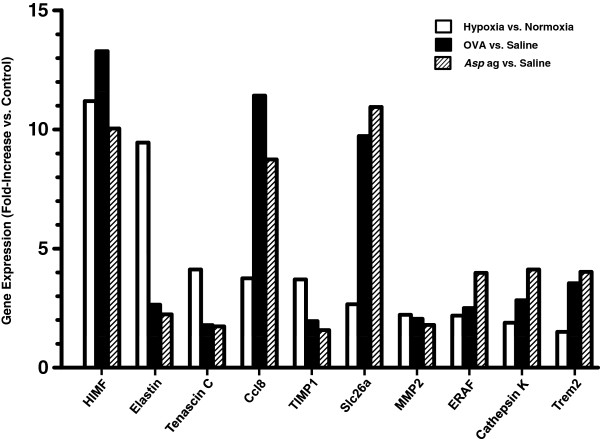
**Genomic analysis of lung tissue from mice exposed to chronic hypoxia and antigen challenge.** Lung tissue samples from normoxia (4 days, 20.8% O_2_, hypoxia (4 days, 10.0% O_2_), saline-challenged, Ova-challenged, and *Asp* ag-challenged mice were processed for cDNA microanalysis. Data are displayed as the mean of the fold-increase versus simultaneous control (normoxia or saline-challenge). n ≥ 3 mice for each group. HIMF: hypoxia-induced mitogenic factor; Ccl8: chemokine (C-C motif) ligand 8; TIMP1: tissue inhibitor of metalloproteinases 1; Slc26a4: solute carrier family 26, member 4; MMP2: matrix metalloproteinase-2; ERAF: erythroid associated factor; Trem2: triggering receptor expressed on myeloid cells 2.

To determine if there were genes that were mutually regulated by both chronic hypoxia and Th2 inflammation, we performed genomic analysis of RNA isolated from lung tissue from mice exposed to both models. In chronic hypoxia experiments, mice were exposed to either hypoxia (10.0% O_2_) or room air (20.8% O_2_) for 4 days. To gain a more complete look at pulmonary Th2 inflammation, we performed genomic analysis on lung tissue from both Ova- and *Asp* ag-challenged mice collected at the conclusion of these experiments. Microarray analysis revealed that 36 transcripts were significantly upregulated in all three murine models (data not shown). Of those genes, ten were related to PH and/or pulmonary injury (Figure 4). Interestingly, of these ten transcripts, HIMF was the most consistently upregulated in all experimental groups. Of the ten genes related to PH and/or pulmonary injury, all could potentially be involved in pulmonary vascular remodeling (Figure 4). One such gene was chemokine (C-C motif) ligand 8 (Ccl8, also known as MCP-2), which may be involved in the recruitment of inflammatory cells to the pulmonary vasculature (Table 1). We also observed the upregulation of elastin, tenascin C, tissue inhibitor of matrix metalloproteinase 1 (TIMP1), solute carrier family 26, member 4 (Slc26a4), matrix metalloproteinase 2 (MMP2), erythroid associated factor (ERAF), cathepsin K, and triggering receptor expressed on myeloid cells 2 (Trem2), which are known to be involved in tissue remodeling (Table 1).

**Table 1:** Commonly upregulated genes

**Gene**	**Potential function in PH**	**References**
**HIMF**	endothelial cell proliferation	[10,14,20,21,29]
	smooth muscle cell proliferation	
	vasoconstriction	
	inflammation	
**Elastin**	tissue remodeling	[43-45]
**Tenascin C**	smooth muscle cell proliferation	[46-49]
	potential PH biomarker	
**Ccl8**	inflammation	[50]
**TIMP1**	tissue remodeling	[51]
**Slc26a4**	inflammation	[52,53]
	mucus secretion	
	airway hyperreactivity	
**MMP2**	tissue remodeling	[49,54]
	potential PH biomarker	
**ERAF**	potential PH biomarker	[55]
**Cathepsin K**	tissue remodeling	[56,57]
**Trem2**	inflammation	[58]

### Evaluation of HIMF expression

**Figure 5:**
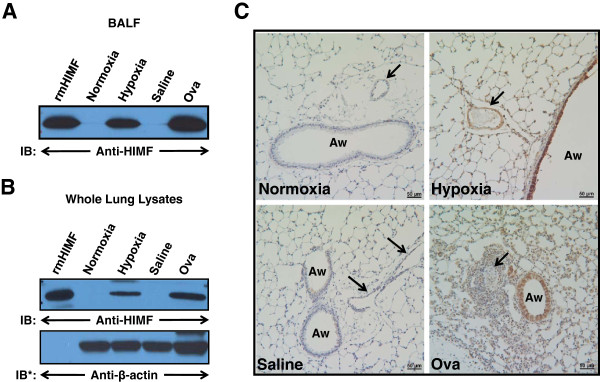
**Expression pattern of hypoxia-induced mitogenic factor (HIMF) following chronic hypoxia or Ova challenge.** (**A**) Bronchioalveolar lavage fluid (BALF) and (**B**) whole-lung homogenates from normoxic (4 days, 20.8% O_2_), hypoxic (4 days, 10.0% O_2_), saline-challenged, and Ova-challenged mice were resolved by 4–20% SDS-PAGE and transferred to nitrocellulose. The blots were probed with rabbit anti-HIMF antibodies and developed using enhanced chemiluminescence. To confirm equal loading and transfer, the blots were stripped and reprobed with β-actin monoclonal antibodies. IB, immunoblot; IB*, immunoblot after stripping. (**C**) Paraffin-embedded lung sections from normoxic, hypoxic, saline-challenged, and Ova-challenged mice were rehydrated and stained with antibodies raised against HIMF (brown). Sections were counterstained with hematoxylin (blue). Aw: airway. Arrows: small pulmonary vessels. Scale bar: 50 μm.

Since HIMF was consistently upregulated in both chronic hypoxia and Th2 inflammation, we therefore examined its expression patterns in both models. HIMF expression was increased in the BALF (Figure 5A) and in whole lung lysates (Figure 5B) from mice exposed to chronic hypoxia (4 days) or Ova challenge. Immunohistochemical analysis of HIMF in these groups revealed differential expression patterns in each (Figure 5C). In chronic hypoxia, HIMF was expressed throughout the lung and was upregulated in airway epithelium, alveolar type II cells, vascular smooth muscle cells, endothelium, and inflammatory cells. It was noticeably upregulated in both peribronchial and intra-alveolar vessels. Ova challenge induced HIMF expression mainly in airway epithelium and inflammatory cells; HIMF expression was noticeably absent from vascular smooth muscle and endothelium in all areas of the lungs of these mice (Figure 5C).

### Cellular localization of HIMF

**Figure 6:**
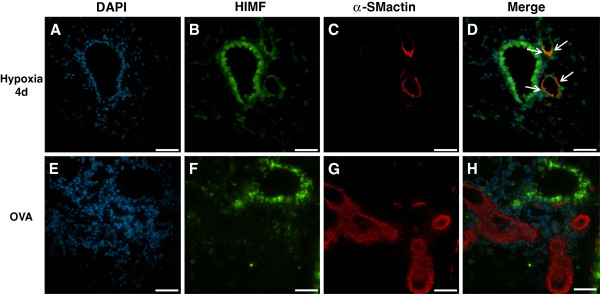
**Co-localization of HIMF with pulmonary vascular smooth muscle.** Paraffin-embedded lung sections from normoxic (4 days, 20.8% O_2_), hypoxic (4 days, 10.0% O_2_), saline-challenged, and Ova-challenged mice were dual stained with rabbit anti-HIMF polyclonal antibody that was visualized by DyLight 488-conjugated goat anti-rabbit IgG antibody (green; **B**, **F**), and mouse anti-α-smooth muscle actin monoclonal antibody that was visualized by DyLight 594-conjugated donkey anti-mouse IgG antibody (red; **C**, **G**). Cell nuclei were counterstained with DAPI (blue; **A**, **E**). The arrows in the merged images indicate co-localization of HIMF and α-smooth muscle actin (**D**, **H**). Scale bar: 50 μm.

**Figure 7:**
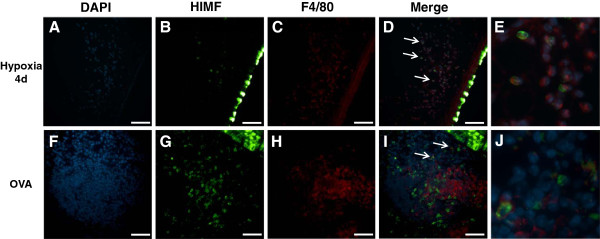
**Co-localization of HIMF with macrophages.** Paraffin-embedded lung sections from normoxic (4 days, 20.8% O_2_), hypoxic (4 days, 10.0% O_2_), saline-challenged, and Ova-challenged mice were dual stained with rabbit anti-HIMF polyclonal antibody that was visualized by DyLight 488-conjugated goat anti-rabbit IgG antibody (green; **B**, **G**), and rat anti-F4/80 monoclonal antibody (macrophages) that was visualized by DyLight 594-conjugated donkey anti-rat IgG antibody (red; **C**, **H**). Cell nuclei were counterstained with DAPI (blue; **A**, **F**). The arrows in the merged images indicate co-localization of HIMF and F4/80 (**D**, **I**). Scale bar: 50 μm. (**E**, **J**) Merged images enlarged to show co-localization.

**Figure 8:**
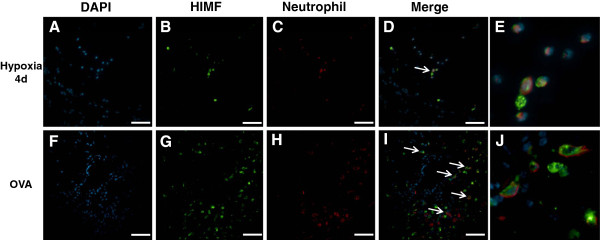
**Co-localization of HIMF with neutrophils.** Paraffin-embedded lung sections from normoxic (4 days, 20.8% O_2_), hypoxic (4 days, 10.0% O_2_), saline-challenged, and Ova-challenged mice were dual stained with rabbit anti-HIMF polyclonal antibody that was visualized by DyLight 488-conjugated goat anti-rabbit IgG antibody (green; **B**, **G**), and rat anti-neutrophil (NIMP-R14) monoclonal antibody (neutrophils) that was visualized by DyLight 594-conjugated donkey anti-rat IgG antibody (red; **C**, **H**). Cell nuclei were counterstained with DAPI (blue; **A**, **F**). The arrows in the merged images indicate co-localization of HIMF and neutrophil marker (NIMP-R14; **D**, **I**). Scale bar: 50 μm. (**E**, **J**) Merged images enlarged to show co-localization.

**Figure 9:**
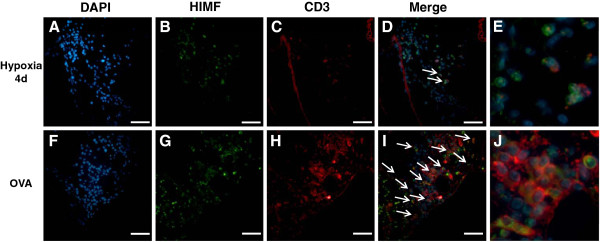
**Co-localization of HIMF with T-cells.** Paraffin-embedded lung sections from normoxic (4 days, 20.8% O_2_), hypoxic (4 days, 10.0% O_2_), saline-challenged, and Ova-challenged mice were dual stained with rabbit anti-HIMF polyclonal antibody that was visualized by DyLight 488-conjugated goat anti-rabbit IgG antibody (green; **B**, **G**), and mouse anti-CD3 monoclonal antibody that was visualized by DyLight 594-conjugated donkey anti-mouse IgG antibody (red; **C**, **H**). Cell nuclei were counterstained with DAPI (blue; **A**, **F**). The arrows in the merged images indicate co-localization of HIMF and CD3 (**D**, **I**). Scale bar: 50 μm. (**E**, **J**) Merged images enlarged to show co-localization.

To determine which specific cell types in the lung express HIMF after chronic hypoxia or Ova challenge and which inflammatory cells are recruited to the lung, we performed immunofluorescence co-localization to compare HIMF expression with the expression of cell-specific markers. Similar to the immunochemistry results, the lungs from mice exposed to chronic hypoxia displayed HIMF-specific immunofluorescence in airway epithelium, pulmonary vasculature, and inflammatory cells; lungs from the Ova-challenged mice had increased HIMF expression in airway epithelium and inflammatory cells (Figures 6, 7, 8 and 9). More specifically, HIMF signal co-localized with α-smooth muscle actin signal following chronic hypoxia (Figure 6D). HIMF and α-smooth muscle actin did not co-localize in the lung sections prepared from Ova-challenged mice (Figure 6H). Interestingly, several HIMF-positive cells were located in close proximity to the vasculature in both chronic hypoxia and Ova challenge models. These HIMF-positive cells were a mixture of macrophages (marker, F4/80; Figure 7), neutrophils (marker, neutrophil marker; Figure 8), and T-cells (marker, CD3; Figure 9). These inflammatory cells were much more prominent around the remodeling vasculature after Ova challenge than after chronic hypoxia. Almost all of the remodeled vessels in Ova-challenged mice were surrounded by a mixture of these HIMF-expressing inflammatory cells, and the overall number of inflammatory cells was large; only a few vessels from the chronic hypoxia model had these HIMF-expressing cells clustered around the vasculature, and the overall number of inflammatory cells was small following this stimulus. Further analysis revealed that B-cells (marker, CD19) and eosinophils (marker, MBP) were also present in the mixture of inflammatory cells surrounding vessels following Ova challenge, but these cells did not express HIMF in our model system (data not shown). Neither cell type was prominently present in the chronic hypoxia model (data not shown).

## Discussion

The current study demonstrates that both chronic hypoxia and repeated airway antigen challenges induce pulmonary vascular remodeling in adult male C57BL/6 mice; however, differences were apparent in the types and extent of remodeling. Exposure to chronic hypoxia for 28 days increased all of the indicators of PH that we examined in this study [mPAP, RV/(LV+S) ratio, vascular remodeling]. In contrast to Ova-challenged mice, hypoxic mice exhibited marked increases in both mPAP and RV/(LV+S) ratio. In addition, mice exposed to chronic hypoxia exhibited an overall neo-muscularization of the pulmonary vessels throughout the entire lung in which both intra-alveolar and peribronchial vessels were thickened. The vessels from this model had the appearance of concentric remodeling. At the 28-day time point, there was little apparent perivascular inflammation. Several recent reports have suggested that Th2 inflammation is involved in the pulmonary vascular remodeling associated with the development of PH [6,13,15-18,59]; therefore, we employed extended Ova-and *Asp* ag-challenge models (3 challenges/week, 4 weeks) to ellicit this response. Analysis of the parameters for PH revealed a slight, but statistically significant increase in mPAP (~1.3-fold) compared to saline control but no change in RV/(LV+S) ratio compared to saline-challenged mice. As expected, extended intranasal Ova challenges produced large amounts of pulmonary inflammation, particularly around the peribronchial vessels, but little new muscularization of peripheral pulmonary vessels; specific analysis of the medial thickness of both intra-alveolar and peribronchial vessels revealed extensive hypertrophy/hyperplasia in only the peribronchial vessels. The remodeling that we observed in peribronchial vessels looked very different from remodeled vessels in the hypoxic mice; the vessels from Ova-challenged mice appeared to have disorganized hypertrophy/hyperplasia. Also, the vessels that appeared to be the most remodeled had the most HIMF-expressing inflammatory cells associated with them.

Several Th2 inflammatory models of pulmonary vascular remodeling have been developed in recent years (e.g., Ova-challenge, *S. mansoni* infection), but these models have limitations regarding the development of PH. As opposed to chronic hypoxia, in which experimental animals consistently display elevated pulmonary and RV pressures, Th2 inflammatory models display little or no pulmonary pressure change [15,19,60]. In the current study, chronic hypoxia induced an almost two-fold increase in both mPAP and RV/(LV+S). Although we did observe a mild increase in the mPAP (~1.3-fold increase) of our Ova-challenged mice, this change was not accompanied by RV hypertrophy. Daley *et al.*[15] reported no changes in right ventricular end systolic pressure in association with antigen challenge. Crosby *et al.*[60] reported extensive pulmonary vascular remodeling associated with a murine model of *S. mansoni* infection, but these animals did not experience significant PH; the authors did suggest that mPAP was higher in those mice that had a greater and more widespread egg burden. In another study on *S. mansoni* infection, Graham *et al.*[19] also reported extensive remodeling, but demonstrated significant RV pressure increases only in IL-13Rα2 knockout mice, not in infected wild-type mice. It is possible that elevated pulmonary pressures were not observed in these models because of the time frames used for these experiments. A follow-up study performed by Crosby *et al.*, [61], demonstrated that *S. mansoni* infection induced pulmonary vascular remodeling, increased RV pressures, as well as RV hypertrophy at 25 weeks following initial infection. These investigators also demonstrated that the severity of the vascular remodeling correlated with the proximity of the *S. mansoni* eggs. Another long-term study performed by Mushaben *et al.*, [62] demonstrated that chronic exposure to house dust mite antigen induced pulmonary vascular remodeling at 7 and 20 weeks of exposures. This study also demonstrated increased RV pressures at 20 weeks, but not at 7 weeks. These studies suggest that it may be possible that a chronic Th2 stimulus may be required for the development of PH in these models.

Immunohistochemical expression patterns of HIMF following either chronic hypoxia or Ova challenge revealed a marked difference between these two models. Most significant among these differences was the consistent expression of HIMF in the vascular endothelium and smooth muscle throughout the lung following chronic hypoxia and the complete absence of HIMF expression in the vasculature following Ova challenge. Hypoxia induced HIMF staining in airway epithelial cells, alveolar type II cells, endothelium, vascular smooth muscle, and inflammatory cells, findings consistent with data that we have previously reported in both mice and rats [20,21] as well as human PH lung [31]. Using double-labeling immunofluorescence microscopy, we also showed that HIMF co-localizes with smooth muscle cells only in the chronic hypoxia model. It is likely that a hypoxic stimulus induces smooth muscle cells within the lung to directly express HIMF, which could initiate the vascular remodeling process. This finding is consistent with our prior work in which we showed that knockdown of HIMF reduced chronic hypoxia–induced vascular remodeling [20]. We did observe that HIMF was expressed in macrophages, T-cells, and neutrophils during chronic hypoxia, but these co-localization events occurred less consistently than did HIMF co-localization with α-smooth muscle actin. After Ova challenge, HIMF was exclusively expressed in airway epithelial cells and inflammatory cells, a finding consistent with those of previous studies that used Ova or *Asp* ag challenge [15,23,27]. The initial study by Holcomb *et al.*[23] demonstrated HIMF expression in alveolar type II cells following aerosolized Ova challenge in adult female BALB/c mice, but no expression in the vascular tissue. The expression pattern of HIMF is very similar in another Th2-dependent model of pulmonary vascular remodeling. Following *S. mansoni* infection, HIMF is also upregulated in areas surrounding the typical granulomas, but not in the remodeled vessels themselves [19]. The lack of HIMF expression in the vascular tissue during inflammation-induced remodeling would suggest that HIMF may be acting on the vasculature through an indirect process. To examine this concept, we identified the inflammatory cells that expressed HIMF after Ova challenge. We found that HIMF was expressed by several different inflammatory cell types, including macrophages, T-cells, and neutrophils, in Ova-challenged mice and that very large numbers of such cells surrounded the remodeling peribronchial blood vessels. The expression of HIMF by macrophages is consistent with previously reported data [23]. To the best of our knowledge, this is the first study to demonstrate that HIMF is expressed in neutrophils and T-cells at the site of inflammation within the lung. At the time point examined, HIMF was not expressed in eosinophils or B-cells. It is possible that HIMF is expressed by these cells [63] at a different time point or in a different mouse strain than those used for this study. We have previously demonstrated that the human homolog to HIMF, RELMβ, is expressed in macrophages and T-cells as well as in vascular cells, plexiform lesions, and myofibroblasts, in patients diagnosed with scleroderma-associated PH [31].

The time course of HIMF expression also differs following hypoxia and antigen challenge. During chronic hypoxia, HIMF mRNA expression is maximal 24 h after initial hypoxic exposure [21]; HIMF protein expression peaks at 4 days of chronic hypoxia and gradually decreases until it reaches baseline levels at approximately 14 days [21]. In the mouse model, the increased HIMF expression correlates with the hyperplastic phase of lung vascular remodeling. This is also consistent with our observations that HIMF is upregulated in the vascular smooth muscle cells that are undergoing cell division [proliferating cell nuclear antigen (PCNA)- and/or Ki67-positive]. Other work from our laboratory supports this hyperplastic role for HIMF, as we have found HIMF to exert a marked shift in human mesenchymal cells to a dividing phenotype [64]. Because HIMF is primarily expressed during the early hyperplastic phase of chronic hypoxia and we have demonstrated that preventing such expression prevents long-term remodeling associated with chronic hypoxia [20], we hypothesize that HIMF acts as an initiating factor for pulmonary vascular remodeling. Lack of HIMF protein within the lungs at this early point of chronic hypoxia reduces the extensive remodeling that would normally result [20]. With Ova challenge, HIMF appears to be consistently upregulated throughout the time period of the model, albeit focal to the area of inflammation. Sun *et al.*[27] has reported increased HIMF expression following an initial 7-day Ova challenge. We and others have demonstrated extensive HIMF expression following extended Ova challenges (3 challenges/week, 4 weeks) [15,22,23,27].

It is currently unknown whether HIMF gene expression is regulated differently through hypoxia and Th2 stimulation or if changes in the two models are parallel. Evidence from sequence analysis and other published work suggests that the transcription factors CCAAT box enhancer binding protein-β (C/EBPβ), hypoxia-inducible factor-1α (HIF-1α), signal transducers and activators of transcription-6 (STAT6), and/or NF-κB may be involved in HIMF gene regulation [21,24,26,65-68]. Stutz *et al.*[26] demonstrated that HIMF itself is activated by the Th2 inflammatory pathway in cultured murine bone marrow cells via IL-4 and IL-13 activation of STAT6 and/or C/EBPβ in its promoter region. Another previous study demonstrated that bleomycin-induced pulmonary HIMF mRNA expression was partially blocked in IL-4 and IL-13 knockout mice [24]. This same study also demonstrated that combined knockout of IL-4 and IL-13 completely blocked HIMF expression in the bleomycin-injured lung. Also, the number of HIMF-positive cells located around remodeled vessels in an *Asp* ag-challenge model was reduced in IL-4 knockout mice compared to that in wild-type controls [15]. In *S. mansoni* infection, HIMF expression is at least partly dependent on IL-13Rα1 [19]. Currently, not much is known about hypoxia-induced regulation of HIMF. In a recent study published by our laboratory, we demonstrated hypoxia-induced HIMF expression in both IL-4 and STAT6 knockout mice [14]. This finding would suggest that Th2 inflammation and hypoxia regulate HIMF expression through different mechanisms. This possibility is consistent with both hypoxia-related and Th2-related transcription factor binding sites in the regulatory regions of the HIMF gene.

We have previously shown that HIMF has mitogenic, angiogenic, anti-apoptotic, vasoconstrictive, and chemokine-like properties both *in vivo* and *in vitro*[10,20,21,28,29,68]. These are key processes involved in the pathogenesis of PH. We have demonstrated that the addition of recombinant HIMF induces a dose-dependent growth response of cultured pulmonary vascular smooth muscle cells [21], and others have shown that intratracheal instillation of HIMF induces proliferation of pulmonary vascular smooth muscle *in vivo*[69]. The addition of recombinant HIMF *in vitro* activates the phosphoinositide 3-kinase (PI-3K) as well as the extracellular signal-regulated kinase 1/2 (ERK1/2) signaling pathways [21,65-67,70-72], both of which are key proliferative pathways. These signaling events occur in isolated primary cultured rodent and human pulmonary vascular smooth muscle cells, illustrating that HIMF acts directly on the cultured cells to induce proliferation; this signaling appears to occur independently of any secondary inflammatory effects that could be induced by HIMF. The differential expression that we have observed may affect the pulmonary vascular remodeling that is observed in each model. It is possible that upregulation of HIMF in both vascular smooth muscle and endothelium may contribute to an autocrine pathway in which HIMF induces cell proliferation within the vasculature. The overexpression of HIMF in the lung vasculature appears to contribute to the overall vascular remodeling of the lung that we are seeing during chronic hypoxia. We have previously shown that AAV-HIMF–induced overexpression of HIMF in the pulmonary vasculature leads to proliferation of both vascular smooth muscle and endothelial cells [20]. During Ova challenge, pulmonary vascular remodeling is localized to the vessels associated with large airways. In this model, large amounts of HIMF are produced and secreted by airway epithelial and inflammatory cells, but no HIMF is expressed in the pulmonary vasculature itself. It is reasonable to suggest that the HIMF secreted into the surrounding areas of the lung affects the local pulmonary vessels and thereby drives the localized remodeling. Daley *et al.*[15] reported that in an *Asp* ag-challenge model, HIMF was expressed in areas surrounding remodeled vessels similar to what we have observed in our Ova-challenge model. The smooth muscle cells in these remodeled vessels also express the markers for cellular proliferation, Ki67 and PCNA [15].

## Conclusions

Our results demonstrate that pulmonary vascular remodeling in mice induced by chronic hypoxia or antigen sensitization and challenge is associated with marked increases in HIMF expression both at the message and protein levels. HIMF was upregulated in the airway epithelium and inflammatory cells in both models, although it was expressed in the vascular smooth muscle of the hypoxia model only. It is certainly possible that the expression of HIMF within the pulmonary vessels themselves may lead to more robust vascular remodeling; therefore, leading to increased pulmonary pressure and right heart hypertrophy. Taken together, our data suggest that HIMF is a key factor in the etiology of both hypoxia- and inflammation-induced models of pulmonary vascular remodeling.

## Abbreviations

PH: Pulmonary hypertension; mPAP: Mean pulmonary artery pressure; HIV: Human immunodeficiency virus; COPD: Chronic obstructive pulmonary disease; PVR: Pulmonary vascular resistance; RV: Right ventricle; LV: Left ventricle; Th1: T-helper 1; Th2: T-helper 2; Ova: Ovalbumin; *Asp* ag: *Aspergillus fumigatus* antigen; *S. mansoni*: *Shistosoma mansoni*; HIMF: Hypoxia-induced mitogenic factor; FIZZ1: Found in inflammatory zone 1; RELMα: Resistin-like molecule alpha; RELMβ: Resistin-like molecule beta; SDF-1: Stromal cell-derived factor-1; MCP-1: Monocyte chemoattractant protein-1; IL-4: Interleukin-4; IL-6: Interleukin-6; IL-13: Interleukin-13; HHV8: Human herpes virus 8; BALF: Bronchalveolar lavage fluid; H&E: Hematoxylin & eosin; NM: Non-muscular; PM: Partially-muscular; FM: Fully-muscular; %MT: % of medial thickness; PB: Peri-bronchial; IA: Intra-alveolar; HRP: Horseradish peroxidase; TBS-T: Tris-buffered saline with 0.1% Tween 20; DAB: 3,3'-diaminobenzidine; SEM: Standard error of mean; PCNA: Proliferating cell nuclear antigen; DAPI: 4’,6’-diamidino-2-phenylindole dilactate; GSMA: Gene Set Matrix Analysis; C/EBPβ: CCAAT box enhancer binding protein-β; HIF-1α: Hypoxia-inducible factor-1α; STAT6: Signal transducers and activators of transcription-6; PI-3K: Phosphoinositide 3-kinase; ERK: Extracellular signal-regulated kinase.

## Competing interests

The author(s) declare that they have no competing interests.

## Authors’ contributions

The experiments were conceived and designed by DJA and RAJ. The experiments were carried out by DJA, QS, KYK, CF, JTS, AP, CC, and HE. DJA, KYK, CF, CC, and RAJ analyzed the data. RAJ contributed reagents/materials/analysis tools for the experiments. DJA, KYK, and RAJ were involved in the writing of the manuscript. All authors have read and approve of this manuscript.
